# NAVIGATING DEMENTIA CAREGIVING AFTER HOSPITALIZATION: CARE PARTNER EXPERIENCES

**DOI:** 10.1093/geroni/igae098.1360

**Published:** 2024-12-31

**Authors:** Ashley Kuzmik, Jane Flanagan

**Affiliations:** Chair; Discussant

## Abstract

Hospitalization and the post-acute transitional period are particularly vulnerable, yet largely underexamined period, in the person living with dementia and their family care partners. The care receivers’ increased care dependency and complex medical and psychosocial needs compound the chronic stress of care partners, and demand new and intensified levels of preparedness. This symposium examines the experiences of care partners and the factors impacting their preparedness and affective responses during transition from hospital to home, using data from the Family-centered Function-Focused Care (Fam-FFC) trial. The first presentation will examine the mediating effects of patient clinical factors on the relationship between care partner preparedness and desire to seek long-term care admission at the time of hospital discharge. Next, the needs for preparedness during transition from hospitalization, as reported by care partners will be discussed. The third paper will describe care partner characteristics that influence care partner preparedness at hospital discharge, and two- and six-months post-discharge. The final presentation will report on the mediational relationship among behavioral and psychological symptoms of dementia (BPSD), associated care partner distress, and care partner adverse outcomes from hospital discharge to six-month follow-up. Concluding the symposium, we will engage in a discussion to address unanswered questions, propose future research directions, and explore the broader implications of the findings for policy and practice.

